# Dataset on assessment of River Yamuna, Delhi, India using indexing approach

**DOI:** 10.1016/j.dib.2018.11.130

**Published:** 2018-11-29

**Authors:** Anshu Yadav, Vinita Khandegar

**Affiliations:** University School of Chemical Technology Guru Gobind Singh Indraprastha University, Dwarka, New Delhi 110078, India

**Keywords:** Heavy metals, Yamuna River, Delhi, Assessment, Physicochemical

## Abstract

The objective of the present study is to investigate the status of pollution load in River Yamuna, Delhi.

The 13 sites for sampling, spread through the Delhi stretch of Yamuna, starting from the Wazirabad barrage till the Okhla barrage has been selected. Physicochemical parameters such as pH, temperature, DO (Dissolved oxygen), TDS (Total dissolved solids), salinity and conductivity were determined. The concentration of heavy metals (Cd, Ni, Zn, Fe, Cu, Pb, and Cr,) were assessed and found to be (0.03, 0.025, 1.365, 6.175, 0.08, 0.02, and 0.03) respectively. Varying concentration of heavy metals was found due to the widespread discharge of industrial effluents into the river. The overall mean concentration of heavy metals was observed in the following order Fe > Zn > Cu > Ni > Cr > Pb > Cd. It can be concluded that our study area as a whole is critically polluted in terms of mean Fe concentration (6.175 mg/L) due to pollutant load from various anthropogenic activities and need treatment before further use. This dataset is beneficial for policymakers, and researchers in the field of River Yamuna water quality management.

**Specifications table**

**Table:** 

Subject area	Environmental Science
More specific subject area	Water monitoring, quality
Type of data	Table and Figure
How data was acquired	All samples were analyzed according to the Standard Methods for Examination of Water and Wastewater. Assessment parameter includes pH, Temperature, Turbidity, Salinity, electrical conductivity (EC), Total dissolved solids (TDS), and Dissolved oxygen (DO) and measured by water analysis Kit (NPC363D, India). Heavy metals were measured using AAS at FICCI, Research and analysis Centre, India.
Data format	Raw, analyzed
Experimental factor	13 sampling sites were selected on the basis of pollution load. Samples were collected in polyethylene bottles and stored in a dark place at room temperature until the metal analysis performed.
Experimental features	7 heavy metals and 7 physicochemical parameters were measured in the River Yamuna water.
Data source location	River Yamuna, New Delhi, India
Data accessibility	This article contains all the dataset

**Value of the data**

• The stretch area between Wazirabad barrage to downstream Okhla barrage is less than 2% of the entire river stretch but it receives around 70% of the total pollution load that received by the river causing sever pollution.
• Water quality assessed in this stretch at 13 locations i.e. Wazirabad Barrage, Boat Club, Kashmere Gate, Nigam Bodh Ghat, Yamuna Ghat, Old Iron Bridge, Geeta Colony, Rajghat, ITO, Pragati Thermal Power Plant (TPP), Akshardham, Sarai Kale Khan and Okhla Barrage
• DO in the river depletes significantly after Wazirabad barrage and remain critical in remaining part of the studied river stretch. The value of this parameter from Wazirabad to Okhla barrage, after joining Shahdara drain was observed in the range of 0.5–3.6 mg/l which reflects that DO is always violating the prescribed standard of 5.0 mg/l.
• Heavy metal pollution Index (HPI) method, is an effective tool to characterize the surface water pollution, and show the composite influence of individual heavy metal on the overall quality of water.
• HPI calculated is far above the critical index limit of 100, that indicates the water is critically polluted with respect to heavy metals. The other reason is of deterioration of Yamuna River water quality in Delhi stretch especially after Wazirabad barrage is due to unabated discharges of wastewater predominantly from domestic sources into the river.

## 1 Data

**Fig. 1:**
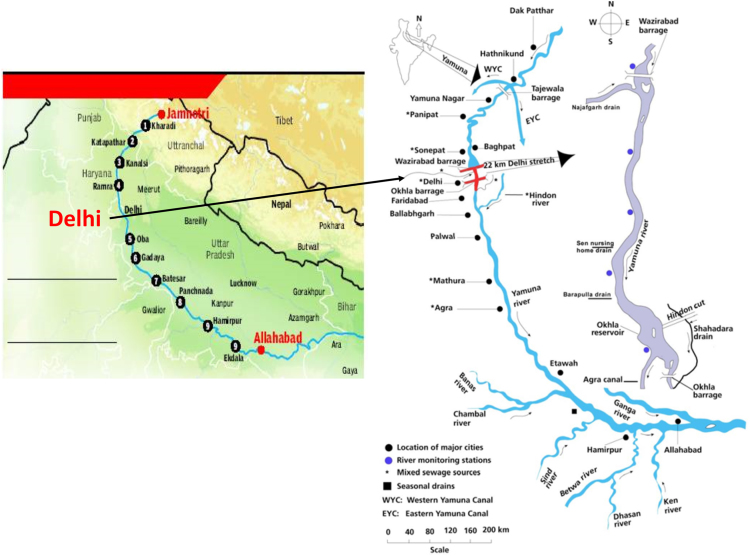
Map of River Yamuna and sampling locations.

**Fig. 2:**
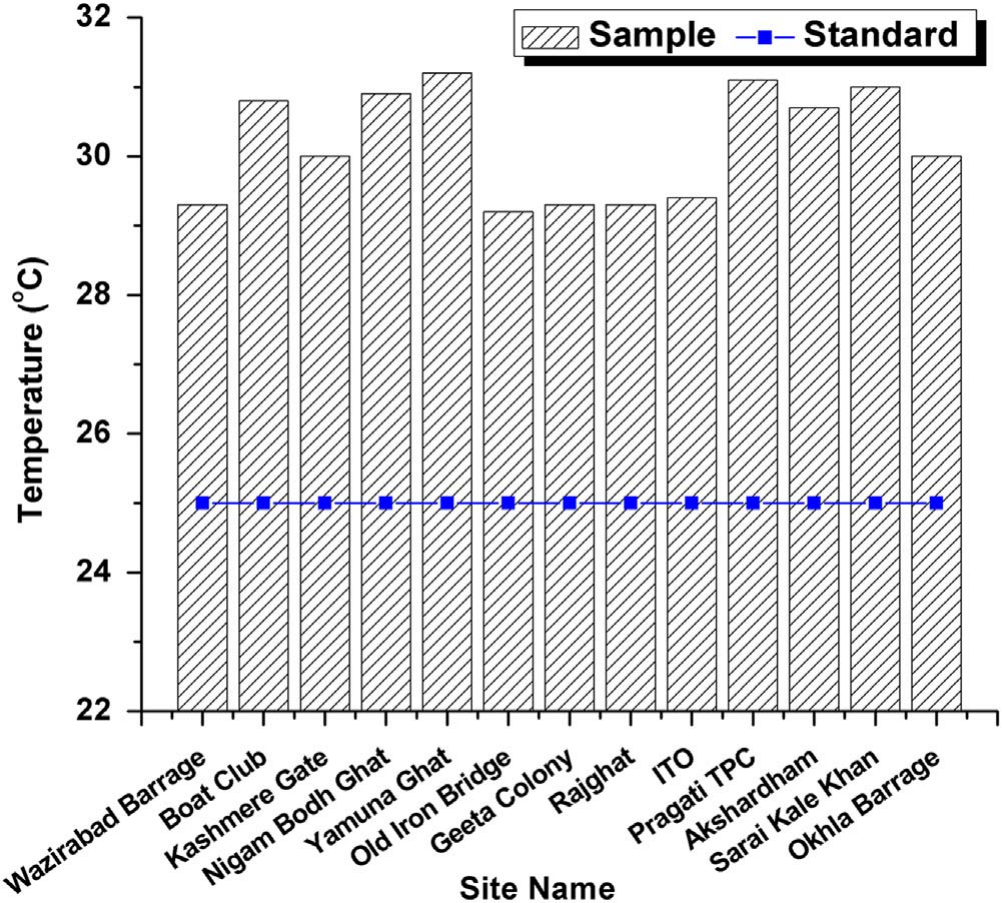
Temperature variation in all 13 samples and BIS standards.

**Fig. 3:**
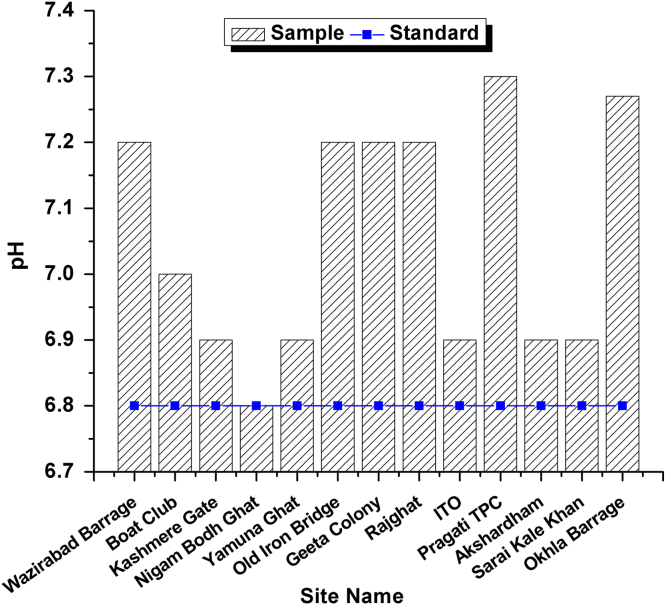
pH Variation in all 13 samples and BIS standards.

**Fig. 4:**
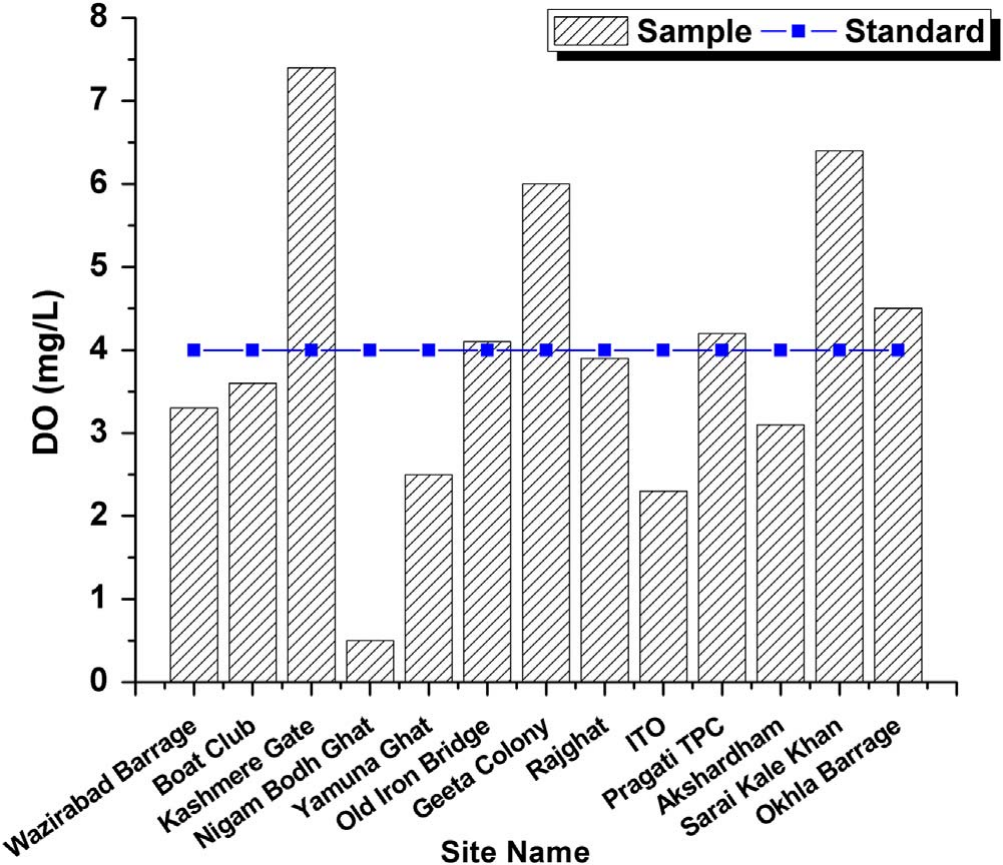
DO Variation in all 13 samples and BIS standards.

**Fig. 5:**
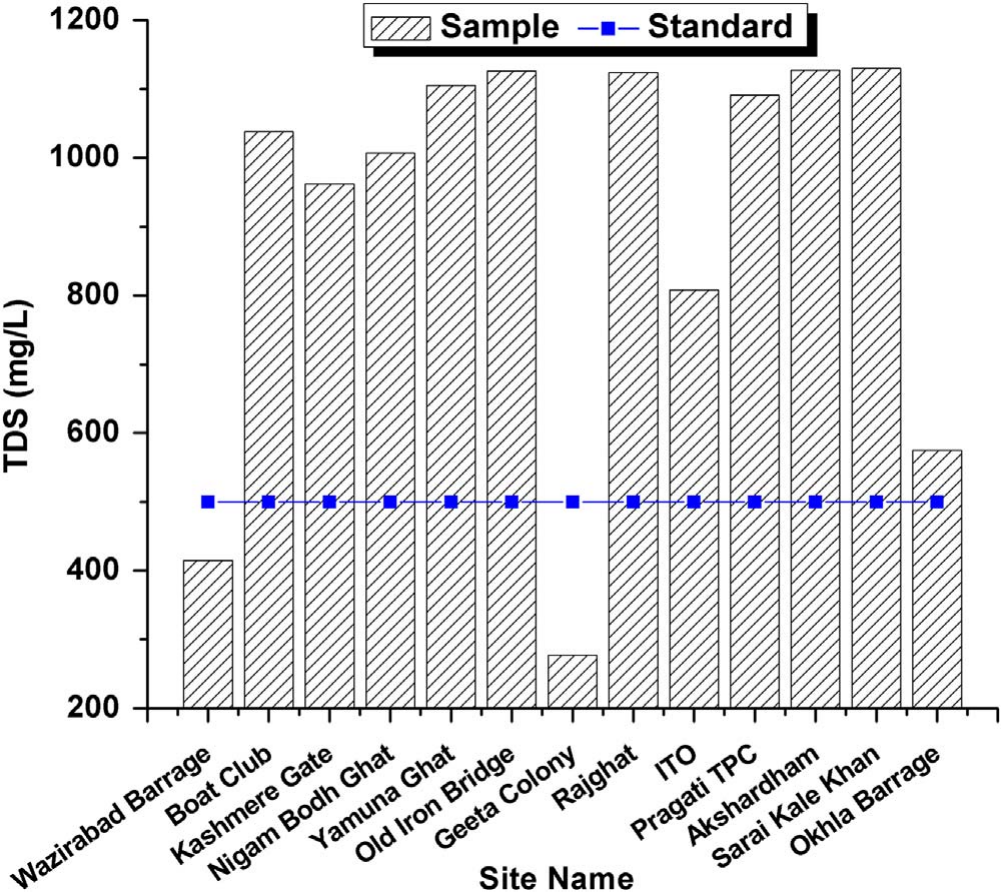
TDS Variation in all 13 samples and BIS standards.

**Fig. 6:**
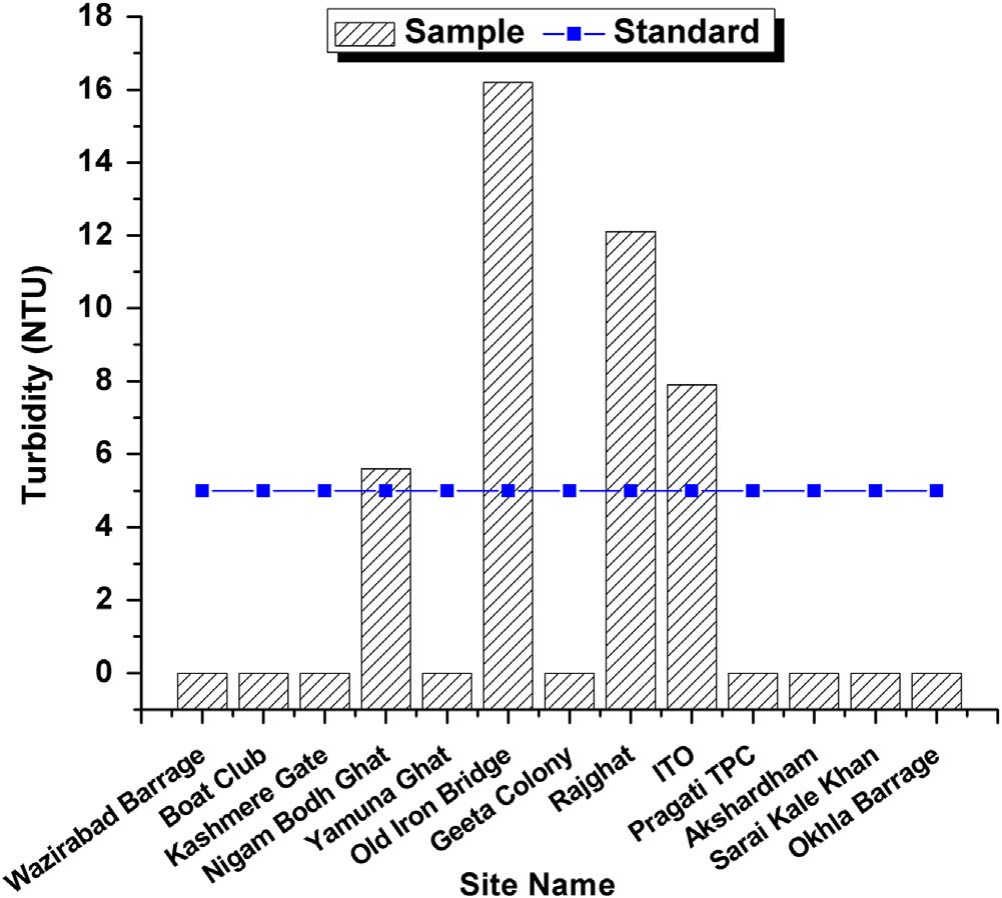
Turbidity Variation in all 13 samples and BIS standards.

**Fig. 7:**
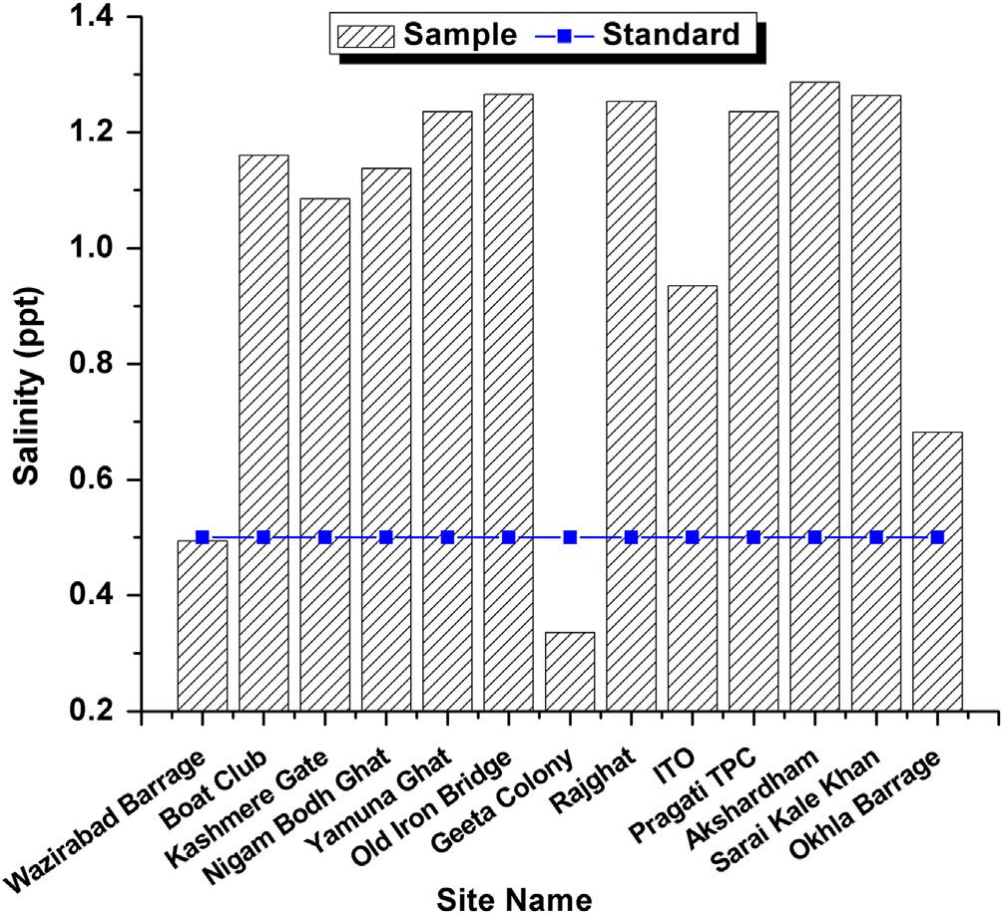
Salinity Variation in all 13 samples and BIS standards.

**Fig. 8:**
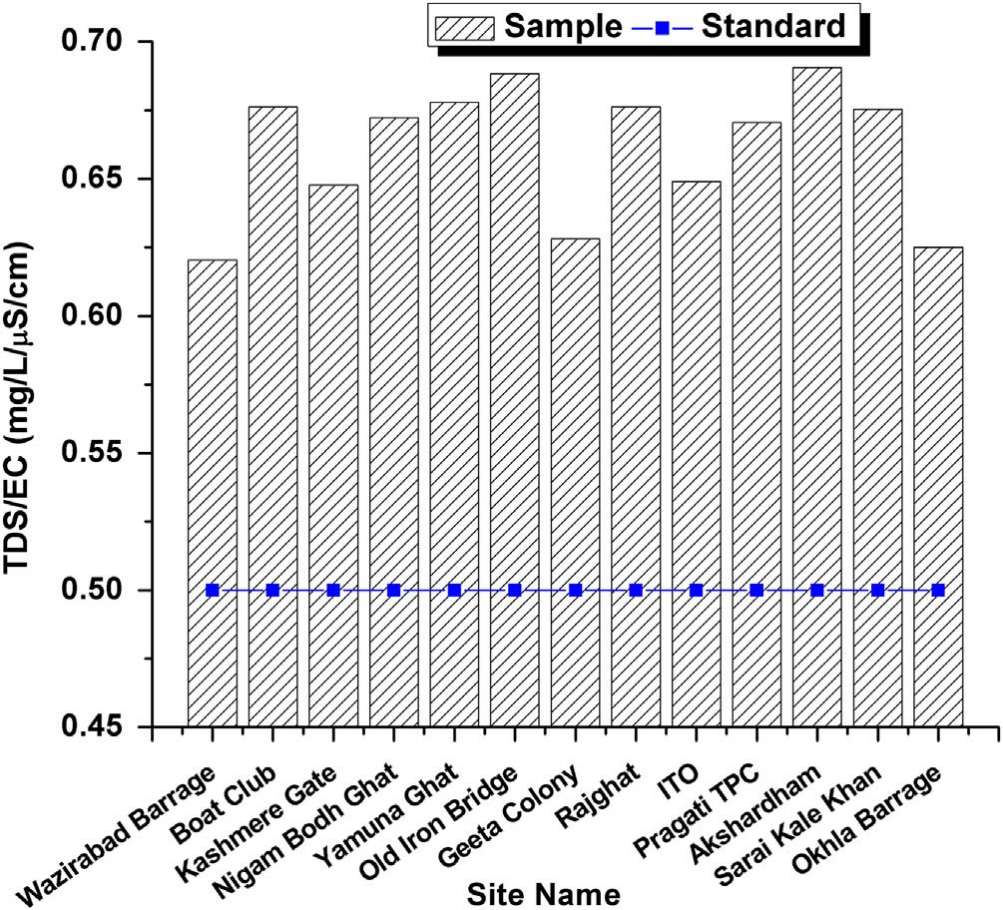
TDS/EC Variation in all 13 samples and BIS standards.

**Fig. 9:**
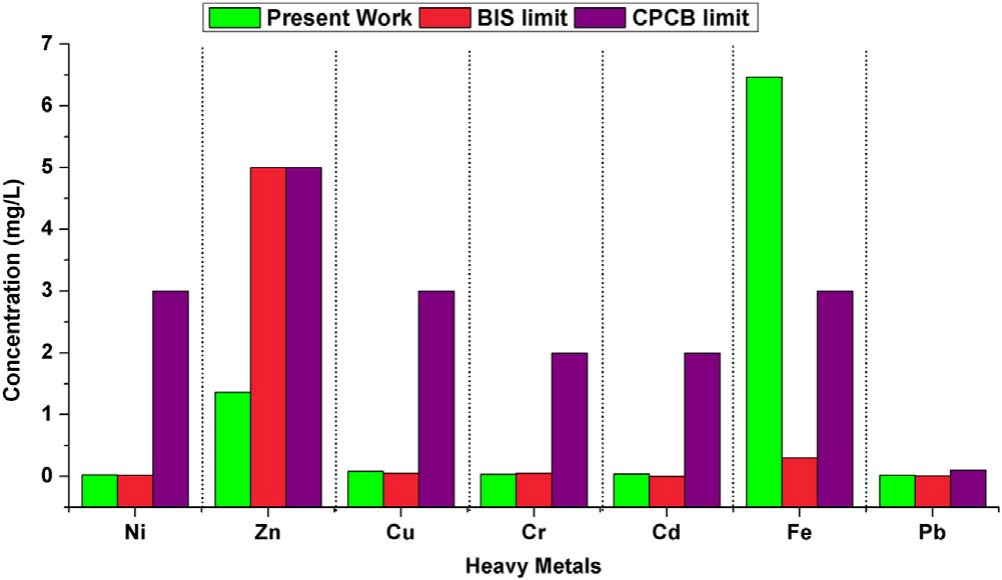
Heavy metals variation in all 13 samples and BIS standards.

This dataset contains 4 Tables and 9 Figures that represent pollution load in River Yamuna, Delhi, India. Fig. 1 and Table 1 shows the sampling points of the study area. Table 2 and Fig. 2, Fig. 3, Fig. 4, Fig. 5, Fig. 6, Fig. 7, Fig. 8 shows the physicochemical characterization determined using standard methodologies [1]. Table 3 and Fig. 9 shows the mean heavy metal concentration and limits prescribed by World Health Organization, Central pollution control Board [4] and Bureau of Indian Standards [2], [3]. Heavy metal pollution index and correlation coefficient of the samples are shown in Table 4 and Table 5 respectively.

**Table 1:** Details of 13 samples collected from River Yamuna, Delhi, India.

**Sample No.**	**Sample location**
1	Wazirabad Barrage
2	Boat Club
3	Kashmere Gate
4	Nigam Bodh Ghat
5	Yamuna Ghat
6	Old Iron Bridge
7	Geeta Colony
8	Rajghat
9	ITO
10	Pragati TPC
11	Akshardham
12	Sarai Kale Khan
13	Okhla Barrage

**Table 2:** Physicochemical analysis of collected samples.

**Site No.**	**Sample location**	**T (**^**o**^C)	**pH**	**Turbidity (NTU)**	**EC (µS/cm)**	**Salinity (ppt)**	**DO (mg/L)**	**TDS (mg/L)**
1	Wazirabad Barrage	29.3	7.2	1.1	669	0.494	3.3	415
2	Boat Club	30.8	7	1.3	1535	1.16	3.6	1038
3	Kashmere Gate	30	6.9	1.5	1485	1.085	7.4	962
4	Nigam Bodh Ghat	30.9	6.8	5.6	1498	1.138	0.5	1007
5	Yamuna Ghat	31.2	6.9	2	1630	1.236	2.5	1105
6	Old Iron Bridge	29.2	7.2	16.2	1636	1.266	4.1	1126
7	Geeta Colony	29.3	7.2	1.1	441	0.336	6	277
8	Rajghat	29.3	7.2	12.1	1662	1.254	3.9	1124
9	ITO	29.4	6.9	7.9	1245	0.935	2.3	808
10	Pragati TPC	31.1	7.3	1.2	1627	1.236	4.2	1091
11	Akshardham	30.7	6.9	1.1	1632	1.287	3.1	1127
12	Sarai Kale Khan	31	6.9	1.4	1673	1.264	6.4	1130
13	Okhla Barrage	30	7.2	1.3	920	0.682	4.5	575

**Table 3:** Mean concentration of heavy metals.

**Heavy metal**	**Value (mg/L)**	**BIS: 10500 (Drinking water)**	**CPCB (Inland surface water)**	**WHO (Drinking water)**
		**Acceptable limit (mg/L)**	**Permissible limit (mg/L)**	**limit (mg/L)**
**Nickel**	0.0254	0.02	3	0.02
**Zinc**	1.3651	5	5	3
**Copper**	0.0813	0.05	3	2
**Chromium**	0.0352	0.05	2	0.05
**Cadmium**	0.0374	0.003	2	0.003
**Iron**	**6.4672**	0.3	3	0.3
**Lead**	0.0212	0.01	0.1	0.01

**Table 4:** Heavy metal pollution Index.

**Heavy metal**	**Mean Value (mg/L) M**_**i**_	**Permissible limit (mg/L) S**_**i**_	**Desirable limit (mg/L) I**_**i**_	**K**	**Unit weightage W**_**i**_	**Q**_**i**_	Qi*Wi	**HPI**
**Nickel**	0.0254	0.02	–	0.4	0.05	127	6.35	**248.5**
**Zinc**	1.3651	5	15	1.005	4.975124378	136.3	678.3532	
**Copper**	0.0813	0.05	1.5	1.005	0.049751244	97.8	4.86773	
**Chromium**	0.0352	0.05		1	0.05	70.4	3.52	
**Cadmium**	0.0374	0.003		1.02	0.002941176	1246.6	3.666667	
**Iron**	6.4672	0.3		0.99	0.303030303	2155.7	653.2525	
**Lead**	0.0212	0.01		1	0.01	212	2.12	
					**∑W**_**i**_**= 5.44**		**∑W**_i_*Q_**i**_**= 1352.13**

**Table 5:** Pearson correlation coefficient matrix.

	Temperature (^o^C)	TDS (mg/L)	Turbidity (NTU)	EC (µS/cm)	Salinity (ppt)	DO (mg/L)	pH
Temperature (^o^C)	1.00						
TDS (mg/L)	0.45	1.00					
Turbidity (NTU)	-0.45	0.47	1.00				
EC (µS/cm)	0.45	1.00	0.45	1.00			
Salinity (ppt)	0.44	1.00	0.47	1.00	1.00		
DO (mg/L)	-0.40	-0.26	-0.21	-0.25	-0.27	1.00	
pH	-0.74	-0.39	0.29	-0.41	-0.40	0.38	1.00

## 2 Experimental design, materials, and methods

### 2.1 River Yamuna

The river Yamuna is of glacial origin and is the sub-basin of the Ganga river system. Out of the total catchment area of 861,404 km^2^ of the Ganga basin, the Yamuna river and its catchment together contributes to a total of 366,223 km^2^ area (catchment basin area in various states accounts for 345,848 km^2^ and Yamuna river area is 20,375 km^2^), which is 42.5% of total Ganga River Basin and 10.7% of the total geographical landmass of the country reported by CPCB [4].

### 2.2 Sample collection

This work was a cross-sectional study. The basis for selection of sampling locations was the changeability within the river stretch considering the variations in the hydrological regimes, pollution and biodiversity characteristics. The specific study sites for sample collection were identified after carrying out the investigation survey of the study area in January 2018. A total of 13 sites from Longitudes 28°45N to 28°30′N and Latitudes 77°13′E to 77 °21 E were selected including various drains falling into River Yamuna and industrial areas with respect to the location of contaminant sources, the point of sewage discharge and the ease of sampling (see in Fig. 1 and Table 1). The samples were collected using grab sampling technique from midstream of the river wherever possible or from well-mixed zone at all the sampling points from a depth of about 0.3 m in duplicate in 5 L high-grade polyethylene bottles. The sampling bottles used were previously soaked and rinsed in 10% HNO_3_ overnight and the collected unfiltered samples were acidified by adding 2 mL of conc. HNO_3_/L of the sample to avoid precipitation of heavy metals. The bottles were capped tightly and stored at 4 °C to prevented evaporation [5], [6], [7], [8], [9], [10], [11].

### 2.3 Physicochemical analysis

All chemicals used in the analysis of samples were of analytical grade and obtained from Merck, India. De-ionized water was used in various water quality protocols followed in the study. Physicochemical parameters including Turbidity, pH, Temperature, conductivity, TDS, Salinity, DO determined using APHA method and cross checked by water analysis kit.

### 2.4 Heavy metal analysis

Heavy metal determined by following the method reported by Bhardwaj et al. [5]. A 50 mL of well-mixed, acid preserved samples were taken in an acid-washed beaker and 10 mL conc. HNO_3_ was added to it. The mixture was digested on a hot plate at 90 °C till the volume got reduced to 10–20 mL. Final volume was made up to 50 mL by addition of de-ionized water that was followed by filtration using Whatman no. 42 filter paper. The digested filtrates were used for the metal quantification using Atomic Absorption Spectrophotometer (model 4141 make: Electronic Corporation of India Limited), with setting of different characteristic wavelengths of metals using hollow cathode lamps and directly aspirating the digested samples into air–acetylene flame. The instrument was calibrated by analyzing known concentration of heavy metals. Standard solutions (1000 mg/L) procured from Merck were serially diluted to obtain the desired concentrations and for each metal, a multi-point calibration graph was prepared. During the analysis, a blank run was performed after every 10 samples to examine the instrument׳s performance for minimization of any errors. The concentrations of each heavy metal in every sample were determined three times, and the results were expressed as the mean concentration of heavy metal in the given sample.

### 2.5 Data evaluation

The aggregate influence of individual heavy metal on the overall quality of River Yamuna water was calculated using Heavy metal pollution index (HPI). Various researchers [5], [8], [9] reported that, HPI is a rating approach that assigns weightage (W_i_) to every parameter, reflecting the relative importance of individual quality considerations in a composite way or Wi can be assessed by making values inversely proportional to the recommended standard (S_i_) for the corresponding parameter. The value of HPI lies between zero and one. The highest tolerant value for drinking water (S_i_) refers to the maximum allowable concentration in drinking water in the absence of any alternate water source. The maximum desirable value (I_i_) indicates the standard limits for the same parameters in drinking water. For computing HPI, the Bureau of Indian Standards (BIS) for drinking water for each heavy metal (chemical parameter) in g/L was considered.

Mohan et al. (1996) reported the HPI as follows:

(1) $$\mathrm{HPI}=\frac{\sum_{i=1}^{n}W_{i}Q_{i}}{{\sum_{i=1}^{n}W_{i}}_{i}}$$

where, Q_i_ is the sub-index of the i^th^ parameter and W_i_ is the unit weightage of the i^th^ parameter and n is the considered parameters in the analysis. The unit weightage (Wi) of the parameter is calculated using Eq. (2), where, S_i_ the maximum allowable recommended standard for i^th^ parameter and k is the constant of proportionality. The sub-index (Qi) of the parameter is calculated using Eq. (3)

(2) $$W_{i}=\frac{k}{S_{i}}$$

(3) $$Q_{i}=\sum_{i=1}^{n}\frac{{M_{i}-I}_{i}}{{S_{i}-I}_{i}}$$

## References

[1] APHA, Standard Methods for the Examination of Water and Waste Water, 1995. 10.2105/ajph.56.3.387 PMC1256912 5948695

[2] WHO, Guidelines for drinking water quality, 2nd Ed. 1: 188, 1993.

[3] BIS, Bureau of Indian Standards 10500 Indian Standard drinking water specification, 1991.

[4] CPCB, Central Pollution Control Board, Report on Water Quality Status of Yamuna River, 2006.

[5] Bhardwaj R., Gupta A., Garg J.K. (2017). Evaluation of heavy metal contamination using environmetrics and indexing approach for River Yamuna, Delhi stretch, India. Water Sci..

[6] Sehgal M., Garg A., Suresh R., Dagar P. (2012). Heavy metal contamination in the Delhi segment of Yamuna basin. Environ. Monit. Assess..

[7] Singh V.K., Singh K.P., Mohan D. (2005). Status of heavy metals in water and bed sediments of River Gomti-a tributary of the Ganga River, India. Environ. Monit. Assess..

[8] Horton R.K. (1965). An index-number system for rating water quality. J. Water Pollut. Control Fed..

[9] Mohan S.V., Nithila P., Reddy S.J. (1996). Estimation of heavy metal in drinking water and development of heavy metal pollution index. J. Environ. Sci. Health.

[10] Ramavandi B., Dobaradaran S., Asgari G., Masoumbeigi H. (2013). High potential for the formation of haloacetic acids in the Karoon River water in Iran. Environ. Monit. Assess..

[11] Seid-Mohammadi A., Roshanaei G., Asgari G. (2014). Heavy metals concentration in vegetables irrigated with contaminated and fresh water and estimation of their daily intakes in suburb areas of Hamadan, Iran. J. Res. Health Sci..

