# Supplementary material for: Dataset on assessment of River Yamuna, Delhi, India using indexing approach
Source: Data Brief. 2018 Nov 29;22:1–10. doi: 10.1016/j.dib.2018.11.130 (PMC6293046; doi:10.1016/j.dib.2018.11.130)
Supplement: Supplementary file 1 — Supplementary material [file mmc1.pdf]

Conflict of Interest

The authors have declared no conflict of interest

Corresponding author

Name: Dr. Vinita Khandegar (Assistant Professor)
Affiliation: University School of Chemical Technology
Guru Gobind Singh Indraprastha University, Dwarka New Delhi-110078, India
